# Vampire Bats and Rabies: Toward an Ecological Solution to a Public Health Problem

**DOI:** 10.1371/journal.pntd.0002867

**Published:** 2014-06-19

**Authors:** Benjamin Stoner-Duncan, Daniel G. Streicker, Christopher M. Tedeschi

**Affiliations:** 1 College of Physicians and Surgeons, Department of Medicine, Columbia University, New York, New York, United States of America; 2 Institute of Biodiversity, Animal Health and Comparative Medicine, University of Glasgow, Glasgow, Scotland, United Kingdom; 3 Odum School of Ecology, University of Georgia, Athens, Georgia, United States of America; London School of Hygiene and Tropical Medicine, United Kingdom

## The Neglected Host of an Already Neglected Disease

**Figure 1:**
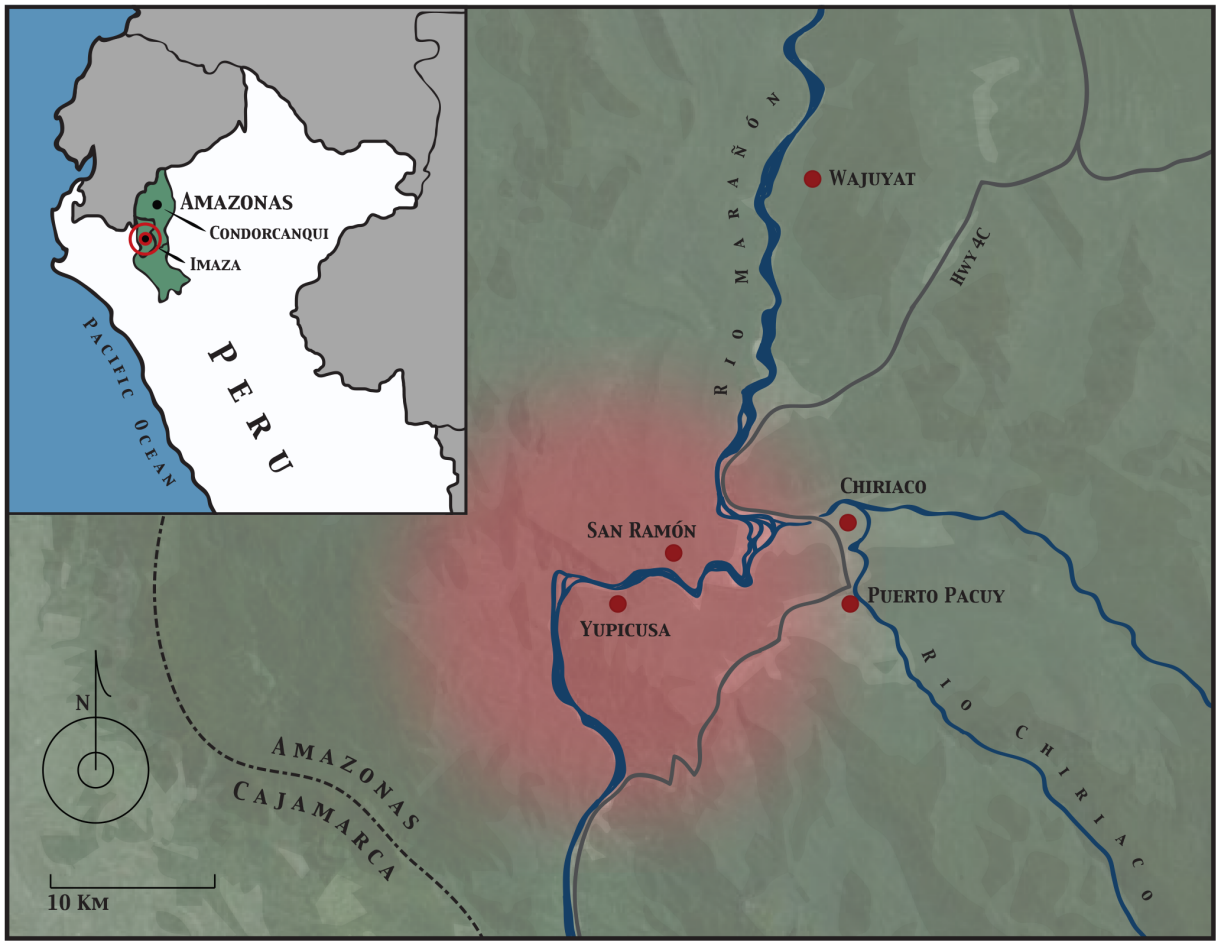
Map of the 2011 outbreak area in the district of Imaza, province of Bagua, department of Amazonas, Peru. The red shaded area highlights the epicenter of the outbreak in the village of Yupicusa. All marked villages have reported recent cases of rabies in humans and/or livestock.

**Figure 2:**
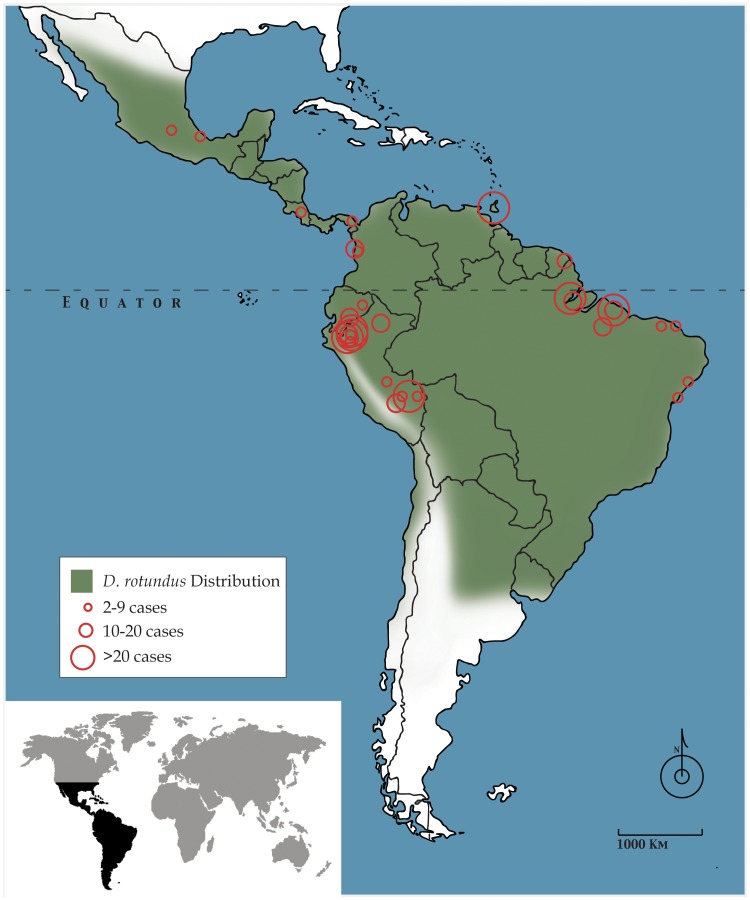
The scope of the problem. Map of Latin America showing the range of *D. rotundus* [20] and reported rabies outbreaks attributed to vampire bat bites [2], [21]–[23]. Aside from a single 1929 outbreak in Trinidad, dates span from 1975 to 2011, with most outbreaks occurring since 1990. Note the high density of outbreaks in northern Peru, department of Amazonas. Sporadic human cases and widespread livestock cases are also reported throughout the range of *D. rotundus*.

In the first half of 2011, 21 school-age children and two adults died of rabies transmitted by the common vampire bat (*Desmodus rotundus*) in and around the small rural village of Yupicusa in the Peruvian Amazon (Figure 1) [1]. This is only one of many such outbreaks occurring throughout the greater Amazon Basin (Figure 2), which, despite efforts at increasing education, vaccination, and bat population control, seem to have escalated over the last three decades—a timeline concurrent with major social and ecological changes in the area [2]. The remote and impoverished nature of communities affected by these outbreaks and the unique niche of vampire bats in a changing socioecological landscape create challenges beyond those faced in previous rabies control efforts and require new strategies to address this public health menace through ecosystem-level intervention. Here we examine this complex system and offer perspectives from a field expedition to Imaza following the 2011 outbreak.

Although the distribution of *D. rotundus* covers most of Latin America (Figure 2), the 2013 World Health Organization (WHO) Expert Consultation on Rabies gives only passing mention to vampire bats, primarily emphasizing the likelihood that infection from this reservoir is underreported. The knowledge gaps highlighted by this comprehensive report allow the perpetuation of untested control strategies and limit effective responses to the reemergence of rabies in countries that have largely eliminated the virus from domestic dog populations [3]. Vampire bats remain a holdout on the global stage of rabies control, the neglected host of an already neglected disease.

## Difficulties with Preventing Human Rabies

The communities affected by vampire bat–transmitted rabies are generally remote, riverine villages with limited access to vaccines and healthcare. Housing typically consists of open-air dwellings, providing no barriers to vampire bat attacks, which can be shockingly common [4]. Mosquito netting is used in some villages, but previous reports have called their efficacy into question [5], and anecdotal reports following the outbreak in Yupicusa suggested lapses in use, particularly by children, who dislike the sensation of sleeping under netting.

The unpredictable nature of outbreaks poses further challenges to prevention, since vigilance can lapse during halcyon times. As a disturbing consequence, vaccination campaigns typically occur only in reaction to local human mortalities. Following the outbreak in Yupicusa, a reactive campaign successfully vaccinated all children and many adults in the village with a standard three-dose “preexposure” course to protect both uninfected and exposed, presymptomatic persons. The duration of immunity provided by this vaccination regime alone is uncertain, and the two additional booster doses recommended following subsequent exposures are currently unavailable to community members. This further highlights the need to develop vaccination recommendations and access for individuals with chronic exposure to bats.

Local beliefs about disease etiology represent another barrier to rabies control in the Amazon. While regional outposts of the Peruvian Ministry of Health have undertaken educational campaigns, some communities hold fast to traditional beliefs. As described to us by one village chief, disease, especially one as devastating and mysterious as rabies, is sometimes still ascribed to witchcraft and thought to be curable only by killing the suspected “witch” (often a member of a neighboring village).

Suspicion of vaccinations also confounds public health efforts. In 2012, after a vaccination campaign in reaction to the Yupicusa outbreak, two children died of an undiagnosed febrile illness in the remote area of Condorcanqui to the north of Imaza in Peru. Local inhabitants attributed these deaths to the vaccinations and threatened health workers, forcing them to leave the area (personal communication: discussions with S. Taqui Paz, 2013, Health Center, Chiriaco). Ongoing hostilities have currently halted human vaccination and rabies investigation in that area of Amazonas.

## A Role for Ecological Interventions?

**Figure 3:**
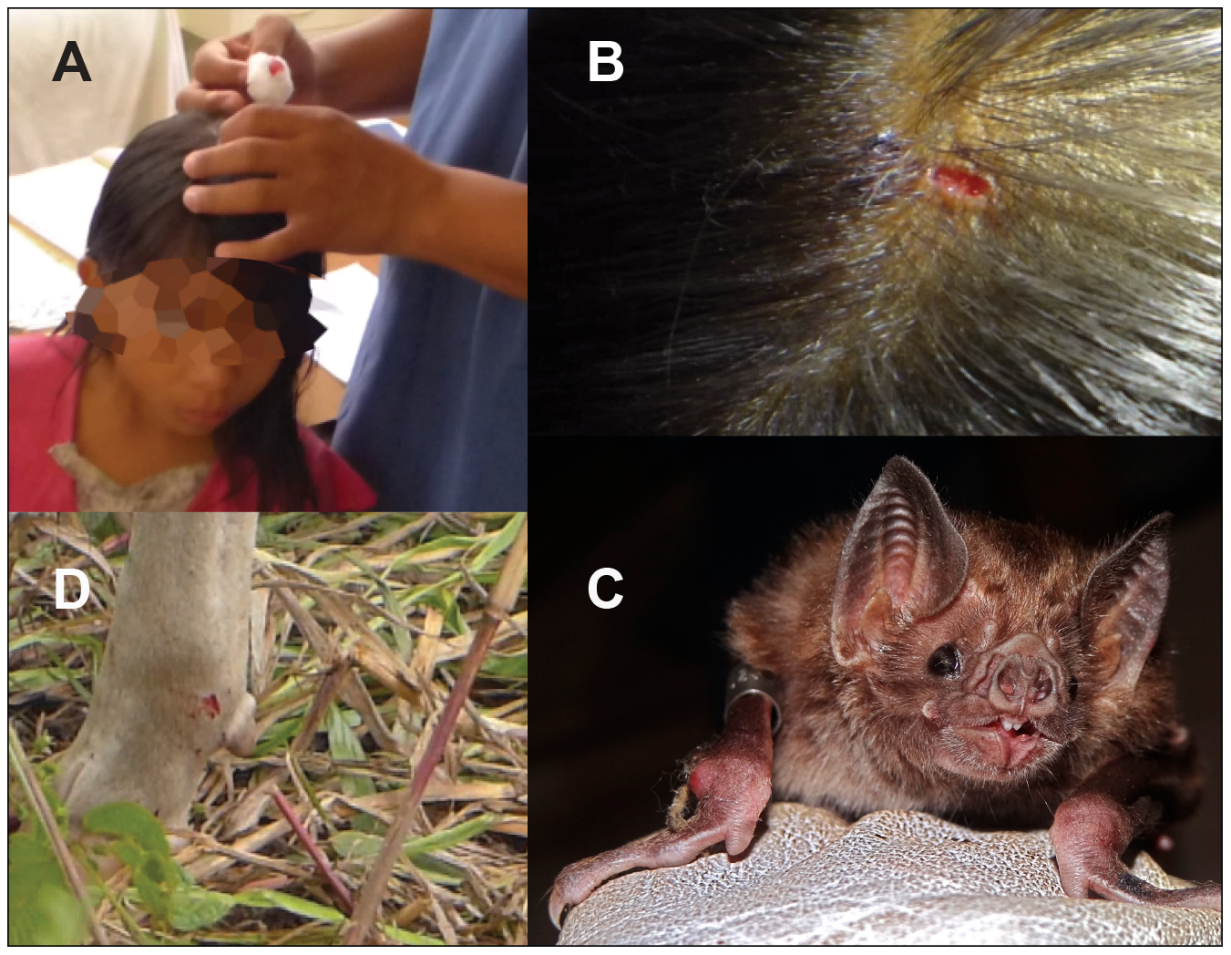
Vampire bat and bites. (A) Acute care in a local health outpost of a young girl bitten by a bat while she slept. (B) Close-up of bite on girl's head showing typical concave lesion. (C) The common vampire bat, *D. rotundus*. The central incisors are used to remove a small patch of skin from prey, and anticoagulants in the saliva prevent clotting while the bat laps the blood meal. This feeding behavior allows for transmission of rabies to prey via saliva. (D) Typical bite on the ankle of a cow.

The ecology of vampire bats as obligate blood feeders provides a unique transmission route for rabies virus (Figure 3) [6]. Thus, human rabies risk is contingent on environmental disturbances that influence bat foraging strategies and infection prevalence. The last 40 years in Amazonian Peru have seen the consolidation of small homesteads into villages, the localized elimination of large wild mammals, and the proliferation of livestock, all of which may have shifted vampire bat feeding from wildlife to anthropogenic sources. Given the difficulty of studying how these complex ecological changes affect bat behavior and rabies transmission, it is understandable that studies of risk factors focus on more tractable elements, such as human demography, living conditions, and age [4], [7]. Simple methods such as the placement of durable netting over openings in a dwelling could address some of these risks but were not practiced in the villages that we visited due to lack of resources and generalized resistance to change. The massive efforts undertaken to vaccinate entire villages should be continued and will likely play a role in decreasing the rabies burden over small areas. However, sustained human vaccination efforts across large spatial scales are logistically infeasible given the complexity of the vaccine injection schedule and the remoteness of many affected communities.

In much of the world, rabies transmitted by domestic dogs and wild *Carnivora* species has been reduced or even eliminated through the vaccination of key reservoir species [8], [9]. Bats, however, pose a special set of barriers to vaccination. Because of their small size, nocturnal behavior, flight ability, secluded roosting, long lifespan, complex reservoir dynamics [10], and widespread, ecologically diverse distribution (Figure 2), mass bat vaccination faces major logistical challenges. Neither vaccine-laden baits (successfully employed in wild carnivores) nor public vaccination campaigns (typical for dog rabies programs) are feasible. Campaigns in which bats are culled using a topical anticoagulant poison remain common in Latin America; however, empirical and theoretical evidence suggests that this strategy may be ineffectual and even counterproductive [11], [12]. Alternative culling practices such as burning or sealing caves kill multiple bat species indiscriminately and must be prevented since many play vital roles in pollination, seed dispersal, and/or insect control, affecting both forest health and agriculture [13]. However, farmers demand bat culls even in the absence of rabies cases because of economic losses from anemia, reduced milk production, and secondary infection in bitten cattle. Future studies should consider sustainable bat population control methods and the role of financial remediation for farmers to generate a partnership balancing financial, public health, and ecological interests.

In the absence of advisable policies for vaccination or culling, the WHO committee concludes that “elimination of bat rabies is therefore not possible at the present time” [3]. Conceptually, however, possibilities exist. New strategies may take advantage of grooming behavior by introducing oral vaccine to the fur of captured bats in much the same way that topical poisons are introduced into caves. One study has shown potential protection by oral vaccination in *D. rotundus* [14], though the optimal application vehicle, dose, and type of vaccine have yet to be determined.

Prey-management strategies might also be considered. The withdrawal of established livestock from villages has been suspected to trigger outbreaks [7], [15]. Vampire bats have been shown through stable isotope analysis to prefer livestock to sylvatic prey, presumably due to abundance and predictability of location [16], [17]. Anecdotal evidence suggests that bats may also prefer livestock to human prey; during our 2-week expedition, we observed only one bite in a human compared to countless livestock bites. If this is the case, vaccinated livestock populations near human habitations could act as a “sink” for vampire bat depredation, potentially reducing human exposure. Taking this concept one step further, an “altruistic” vaccine could be introduced into livestock, thereby inoculating vampire bats as they feed. Production of such a delivery system for vaccination presents a significant pharmaco-immunological challenge; however, an analogous strategy has been used to deliver anticoagulant poisons to bats [18], and similar vaccines exist for malaria control [19].

Lastly, conservation has been a low priority in Amazonia, with many of the natural prey of vampire bats having been displaced decades ago by resource extraction and agricultural expansion. Further study is needed to understand the wildlife feeding preferences of vampire bats, potentially leading to conservation efforts to reestablish historical feeding strategies, thereby reducing human depredation.

## Conclusions

Despite being recognized for over a century as a threat to human and livestock health, vampire bat–transmitted rabies continues to be neglected in terms of research and effective control. Areas of Amazonia, especially in Peru and Brazil, have recorded an increased incidence of rabies outbreaks in humans over the past several decades, despite disease management efforts at the local and national levels. Barriers impeding these efforts include the remote and impoverished nature of communities at greatest risk, finite government resources, and a poor understanding of viral persistence mechanisms in the vampire bat reservoir.

New and creative approaches are needed to address the problem of vampire bat–transmitted rabies in Amazonia. Future research should focus on (1) vampire bat feeding behavior; (2) the potential impacts of ecological change and human interventions on rabies transmission from bats to humans and domestic animals; (3) further description of individual and community risk factors for rabies outbreaks; and ultimately (4) the development of novel delivery systems for rabies vaccination in bats.

## Supporting Information

Video S1 **Footage from recent field work in the outbreak area, Imaza, Peru.**
(MP4)
